# The spatial and temporal properties of the contour erasure effect and perceptual filling-in

**DOI:** 10.1167/jov.25.14.4

**Published:** 2025-12-11

**Authors:** Yih-Shiuan Lin, Chien-Chung Chen, Mark W. Greenlee

**Affiliations:** 1 Institute of Experimental Psychology, University of Regensburg, Regensburg, Germany; 2 Department of Psychology, National Taiwan University, Taipei, Taiwan; 3 Neurobiology and Cognitive Science Center, National Taiwan University, Taipei, Taiwan

**Keywords:** contour erasure, two-alternative forced choice (2AFC), filling-in, spatiotemporal properties

## Abstract

Contour erasure describes the phenomenon that after brief flicker adaptation at the edge of an object, the object disappears and is replaced by the background – highlighting the importance of edges in perceiving a surface. The underlying mechanism remains unknown. The current study investigates the characteristics and functional properties of contour erasure, and its relationship with related phenomena such as perceptual filling-in, forward masking, and contrast adaptation. We used a homogeneous disk as a target, and circles that corresponded to the outline of the target disk as the adapter. Using a two-alternative forced choice (2AFC) paradigm, each trial began with a counterphase flickering adapter, followed by the target randomly presented in one of the two locations. Participants indicated the target location with a button press. The target detection threshold elevation relative to the no adaptation condition was used as an index of the adaptation effect. We manipulated two spatial properties (eccentricity and the adapter size) plus three temporal properties (adapter flickering rate, adaptation duration, and interstimulus interval [ISI]). Results indicated that the adaptation effect increased with eccentricity, flickering rate (plateauing at 6 hertz [Hz]) and adaptation duration, but decreased with longer ISI and for adapter sizes that were larger than the target. The target threshold first increased then decreased as the adapter size decreased from that of the target, indicating a size tuning that is slightly smaller than the target. Our results indicate that contour erasure shares some of the key features of other well-known perceptual phenomena like filling in and contrast adaptation.

## Introduction

Contour erasure refers to the visual phenomenon in which an object vanishes from sight after brief adaptation to its edges. This effect can be easily demonstrated using a contour adapter shaped like the outline of a homogeneous disk. After flickering the contour adapter in counterphase for a few seconds, the subsequently presented disk disappears into the background. This phenomenon was first reported by (Anstis, 2013), who described several key characteristics of the contour erasure effect. First, it does not transfer interocularly, that is, monocular adaptation of the one eye does not transfer to the unadapted eye. Second, it fails to render chromatic objects invisible. Third, it does not affect illusory contours generated by Kanizsa-type inducers. A follow-up study by Anstis and Greenlee (2014) provided further insight by comparing contour erasure to other perceptual filling-in effects. They found that the adapter must be congruent with the object edges in size and shape, and that a contour-only adapter was more effective than a full, blurred disk in making the object disappear. In one condition, a vertical line adapter placed between two halves of a disk with differing contrast levels made the entire disk appear uniformly grey, indicating a long-range interaction induced by contour erasure. These findings suggest that contour erasure likely occurs at an early stage of visual processing, possibly in the magnocellular pathway.

Contour erasure also interacts with other visual phenomena. For example, White's illusion describes the perception of luminance differences when equiluminant gray patches are placed on the black and white stripes of a square-wave grating: the patch on the black stripe appears brighter than the one on the white stripe (White, 1979). This illusion highlights the contextual nature of brightness perception. Betz, Shapley, Wichmann, and Maertens (2015) applied contour erasure to either the edges parallel or orthogonal to the stripes and found that adapting parallel edges slightly enhanced the illusion, whereas adapting orthogonal edges abolished or even reversed it. They concluded that the illusion is primarily determined by orthogonal edge contrast, which is disrupted through contour erasure. In another example, the watercolor illusion involves color spreading from edges into adjacent surfaces (Pinna, Brelstaff & Spillmann, 2001; Pinna, Werner, & Spillmann, 2003). Coia and Crognale (2018) found that contour erasure at the watercolor edges reduced the perceived contrast and saturation of the illusion. These findings suggest that contour erasure can eliminate edge information critical for maintaining surface boundaries. By weakening or removing such boundaries, the effects that rely on edge contrast are diminished. The mechanisms underlying contour erasure remain elusive. Whereas some researchers relate it to contrast adaptation, others compare it to perceptual filling-in.

Contrast adaptation occurs when sensitivity to contrast is reduced after adapting a retinal region to a grating with the same time-averaged luminance, thereby leaving no afterimage. This adaptation reduces sensitivity to test gratings that share spatial frequency and orientation with the adapter (Blakemore & Campbell, 1969; Kelly, 1983; Moradi & Shimojo, 2004; Webster & Mollon, 1993). One form of contrast adaptation is flicker adaptation, where sensitivity to a flickering target is reduced after exposure to a counterphase flickering adapter (Pantle, 1971). Robinson and de Sa (2012) proposed that flicker adaptation involves two mechanisms: an edge mechanism sensitive to spatial contrast, and a surface mechanism responsive to temporal luminance changes without local contrast (Tyler, 1975). They showed that as the adapter's edge became less aligned with the target's edge, adaptation effects diminished, implicating both edge and surface mechanisms. In a second experiment using a flickering annulus, they found that edge-only adaptation produced a modest effect, but stronger adaptation required engagement of both mechanisms. Their findings imply that adapting only the edge of a target can reduce sensitivity, a result echoed in contour erasure studies. A follow-up study (Robinson & de Sa, 2013) using dynamic brightness induction confirmed that adaptation was effective only when the target's edge matched the adapter's edge, reinforcing the role of boundary-specific adaptation.

Despite similarities, contrast adaptation and contour erasure differ in key ways. Contrast adaptation exhibits interocular transfer (Moradi & Shimojo, 2004), whereas contour erasure does not (Anstis, 2013). Furthermore, contrast adaptation can occur with chromatic stimuli (Webster & Mollon, 1993), whereas contour erasure affects only achromatic stimuli, with the exception of illusory color spreading (Anstis, 2013; Coia & Crognale, 2018). When comparing the adaptation duration required to induce both adaptation effects, contour adaptation has typically been studied with shorter flicker exposures (Anstis, 2013; Anstis & Greenlee, 2014). However, although contrast adaptation is often studied with relatively long adaptation durations (Foley & Boynton, 1993), it can also be induced with much shorter exposures, such as sub-second presentations (Pavan, Marotti, & Campana, 2012). Importantly, the contrast adaptation duration primarily influences the recovery time rather than the magnitude of the contrast adaptation effect itself (Greenlee, Georgeson, Magnussen, & Harris, 1991). Therefore, it is not yet clear whether contrast adaptation generally requires longer adaptation periods than contour erasure effect. These differences suggest that distinct neural mechanisms underlie the two phenomena.

Contour erasure has also been compared to perceptual filling-in, in which the visual system “fills in” missing information based on surrounding context (De Weerd & Pessoa, 2003; Komatsu, 2006; Weil & Rees, 2011). The most prominent example is the blind spot, where absent photoreceptor input is seamlessly filled in by surrounding visual features (Ramachandran, 1992; Spillmann, Otte, Hamburger, & Magnussen, 2006). Filling-in also occurs in peripheral fading (Stürzel & Spillmann, 2001; Troxler, 1804) and artificial scotomas (Mihaylov, Manahilov, Simpson, & Strang, 2007; Ramachandran & Gregory, 1991), where blank regions surrounded by flickering noise are perceptually filled in over time. Contour erasure may be conceptualized as a process that accelerates filling-in. Anstis (2013) proposed a two-stage process: slow fading of contours, followed by rapid filling-in from the surround (Spillmann & De Weerd, 2003). However, contour erasure occurs rapidly and selectively for luminance-defined stimuli. The absence of similar effects for textured or patterned objects suggests that contour erasure might involve a distinct type of filling-in.

In the current study, we used contrast increment threshold measurements as an objective method to quantify the effects of contour erasure under different spatial and temporal conditions. In a two-alternative forced choice (2AFC) task, participants identified the location of a briefly presented target after contour erasure.

We manipulated two spatial factors: (1) stimulus eccentricity, and (2) the size of the contour adapter. Eccentricity was included to test whether contour erasure, like scotoma filling-in, is enhanced in peripheral vision (De Weerd, Desimone, & Ungerleider, 1998). Previous research has shown that the adapter must match the target's size to induce contour erasure. However, Anstis and Greenlee (2014) observed that oversized ring adapters made the adapted disk appear smaller, and undersized rings made it appear larger—suggesting long-range interactions. We therefore varied adapter size to investigate this lateral modulation.

We also manipulated three temporal factors: (1) adapter flicker rate, (2) adaptation duration, and (3) interstimulus interval (ISI) between the adapter and the target. Flicker rate helps distinguish whether sustained or transient mechanisms are involved (Kaneko, Giaschi, & Anstis, 2015; Lennie, 1980). Adaptation duration may reveal the time course of the buildup of the effect, and the ISI allows us to examine the recovery curve of contour erasure. Together, these manipulations aim to provide a more comprehensive understanding of the spatial and temporal dynamics of the contour erasure effect.

### Methods

#### Participants

Six participants (aged 23–32 years), all with normal or corrected-to-normal vision, took part in the study. One participant (P0) was one of the authors (Y.S.L.), whereas the remaining five participants (P1–P5) were naïve to the purpose of the study. Participant P0 completed all five experiments. Participant P1 participated in eExperiments 1, 2 (at 5.7 degrees eccentricity), 3, 4, and 5 (at 5.7 degrees). Participant P2 took part in experiments 1 and 3 only. Participant P3 participated in experiments 2 and 5, both at 2.8 degrees eccentricity. Participants P4 and P5 completed experiments 2, 4, and 5, all at 5.7 degrees eccentricity. Participants were recruited from both National Taiwan University and the University of Regensburg. Informed consent was obtained from all participants prior to the study. The experimental protocols were approved by the ethics committee of the University of Regensburg (application number: 19-1591-101) and the Institutional Review Board of National Taiwan University (202001HM013). All procedures adhered to the Declaration of Helsinki. Participants (except P0) received monetary compensation or course credit for their participation.

#### Apparatus

In the experiments conducted at the University of Regensburg, participants viewed stimuli on either a Dell S2417DG 24-inch LCD monitor (2560 × 1440 resolution, 73.8 cd/m² mean luminance) or a Philips 272S1AE LCD monitor (1920 × 1080 resolution, 73.6 cd/m² mean luminance). Both monitors operated at a 60 hertz (Hz) refresh rate and were calibrated using a MINOLTA CS-100 spot photometer. The viewing distance was adjusted so that each pixel subtended 1 arcminute. The experiments were conducted in a dimly lit room.

For experiments at the National Taiwan University, an EIZO EV2456 24-inch LCD monitor (1920 × 1200 resolution, 67.5 cd/m² mean luminance, and 60 Hz refresh rate) was used. This monitor was calibrated with a SpectraScan PR655 Spectroradiometer. The viewing distance was similarly adjusted to ensure that each pixel corresponded to 1 arcminute of visual angle. The experiments were also conducted in a dimly lit room.

### Stimuli and Procedure

**Figure 1.**
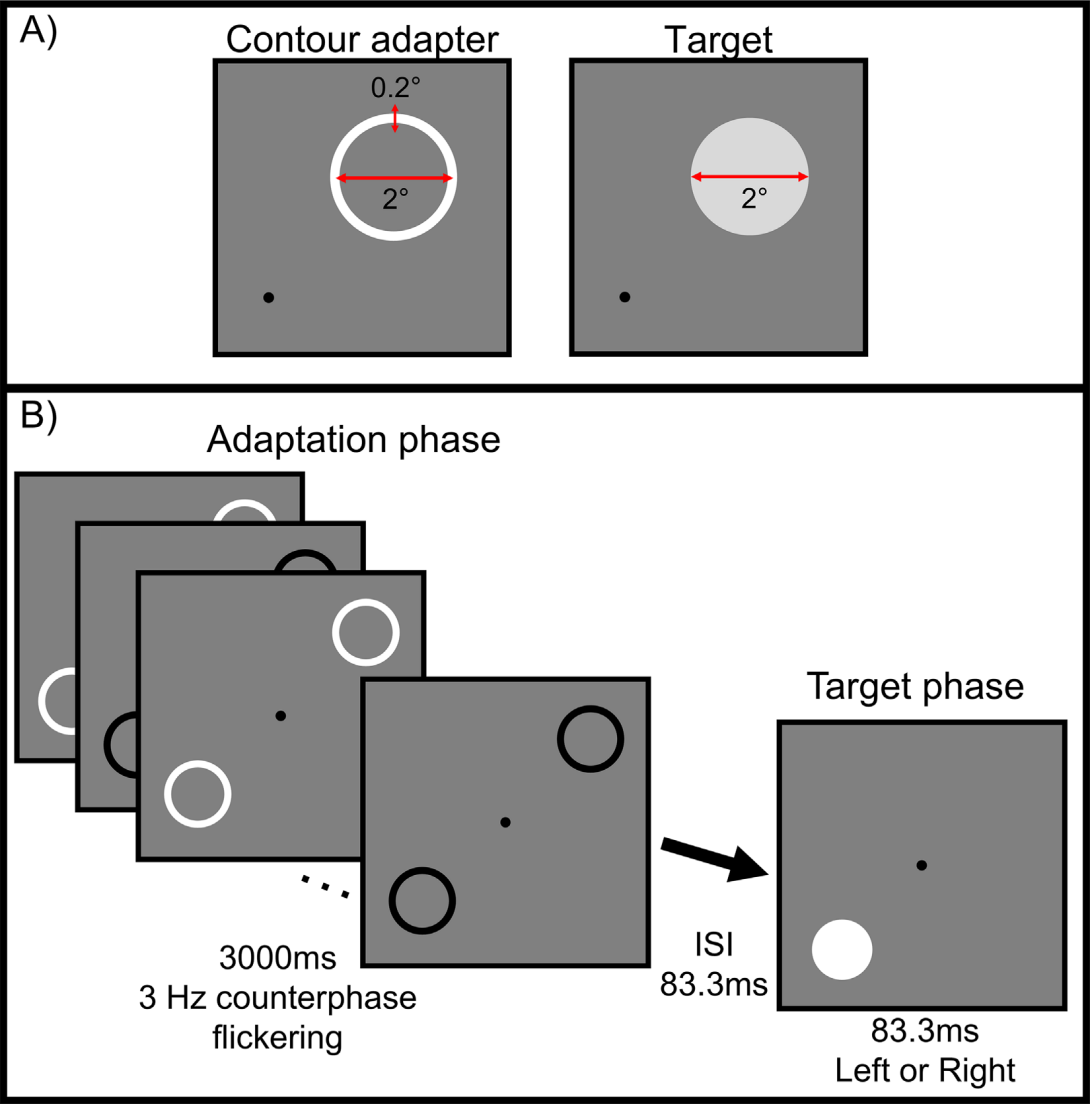
Demonstration of the stimuli and procedure. Panel (**A**) depicts the contour adapter, a 2 degrees diameter high contrast ring of 0.2 degrees width, on the left and the target, a 2 degrees diameter homogeneous disk, on the right. Panel (**B**) shows the procedure of one trial in the 2AFC paradigm, in which two high-contrast contour adapters flickered in counterphase for 3 seconds, followed by a short ISI, then the target randomly at one of the two locations. The participants were to detect the target location and respond by pressing a corresponding key.

Figure 1 illustrates the stimuli and procedure used in the study. The target was a uniform disk subtending 2 degrees of visual angle in diameter, presented against a mean luminance background. Stimulus contrast is defined by Weber contrast (stimulus contrast = [stimulus luminance-background luminance]/background luminance). The target had positive contrast, whereas the adapter consisted of a high-contrast ring with variable diameter and a fixed width of 0.2 degrees, overlapping the edge of the target. The adapter contrast was set at −1 decibels (dB; where 20 dB corresponds to 1 log unit or one order of magnitude), or 89.13%, and flickered between positive and negative polarity during adaptation. Stimuli were presented at eccentricities ranging from 2.8 degrees to 11.3 degrees. All stimuli were generated using MATLAB (MathWorks, Natick, MA, USA) and Psychtoolbox (http://psychtoolbox.org/).

A 2AFC paradigm was used to measure participants’ luminance contrast detection thresholds. Each trial consisted of an adaptation phase followed by a target phase. During the adaptation phase, two flickering contour adapters were presented at 3 Hz for 3 seconds unless otherwise noted. In control conditions (no adaptation), only a fixation point was presented during this phase. During the target phase, a target appeared in one of two spatial locations. Participants indicated the target’s location using keyboard input. Auditory feedback was provided: a high-pitch tone for correct responses and a low-pitch tone for incorrect responses.

To estimate thresholds, we implemented the PSI method, a Bayesian adaptive staircase procedure (Kontsevich & Tyler, 1999), which targeted the 86% correct performance level. The algorithm adjusted the target contrast on each trial based on the participant's performance. An intertrial interval (ITI) of 1.5 seconds followed each response. All participants completed a short practice session prior to the formal experiment.

We manipulated five key factors in five experiments (see, Table 1):

**Table 1.** Summary of the five experiments.

Exp. No.	Manipulated factor	Number of participants	Approx. experimental time (per participant)
1	Eccentricity (2.8 degrees to 11.3 degrees)	3 (P0, P1, P2)	90 mins
2	Adapter size (0.5 degrees to 4 degrees)	5 (P0, P1, P3, P4, P5)	120 mins
3	Adapter flicker rate (1.5 Hz to 10 Hz)	3 (P0, P1, P2)	60 mins
4	Adaptation duration (333 ms to 6000 ms)	4 (P0, P1, P4, P5)	60 mins
5	Interstimulus interval (ISI) (33.3 ms to 2667 ms)	5 (P0, P1, P3, P4, P5)	120 mins

Each of these variables was varied independently, with all other parameters held constant. Each experimental condition was repeated at least three times for each participant.

### Data analysis

Data analysis and visualization were carried out using MATLAB 2024a (MathWorks, Natick, MA, USA) and R version 4.4.2 (R Core Team, 2023). Nonlinear curve fitting was performed using MATLAB’s lsqcurvefit function, which finds the least-squares solution of the free parameters. Statistical tests were conducted using the rstatix and stats packages in R. rstatix provides functions for common statistical tests, effect sizes, and assumption checks, using a tidy and pipe-friendly syntax (Kassambara, 2023). The base stats package includes essential statistical tools such as *t*-tests, ANOVAs, and regressions, and was used for core analyses. Visualization was performed using the ggplot2 package, a plotting package that builds graphics using a layered grammar to create clear and flexible data visualizations (Wickham, 2016). Bayesian inference was carried out following the approach described by Wagenmakers (2007). We estimated the strength of evidence for model effects by converting ANOVA results into approximate Bayes factors using the Bayesian Information Criterion (BIC). The difference of BICs (ΔBIC) between two models provides a quantitative information for model selection. Comparing two ANOVA models with a difference in a particular term(s) provided a test of significance for that term. In general, the greater the deviation from 1 is, the greater the likelihood of a model is over the other (Raftery, 1995). An absolute value of ΔBIC of 0 to 2 indicates weak evidence, 2 to 6 moderate evidence, 6 to 10 strong evidence, and >10 very strong evidence.

## Results

Overall, five experiments were conducted in this study, each focusing on one key characteristic of the contour erasure effect. Although one factor was being varied, the other factors were fixed at a constant level. Whereas one factor was systematically varied, the remaining parameters were held constant. Unless otherwise specified, the standard stimulus parameters were: 3-second adaptation duration, 3 Hz adapter flickering rate, 83.3 ms target presentation time, 83.3 ms ISI, 5.7 degrees eccentricity, and 2 degrees diameter for both the adapter and the target. The raw threshold data of each participant are provided in the supplementary files.

### Experiment 1: Effect of eccentricity (spatial factor 1)

**Figure 2.**
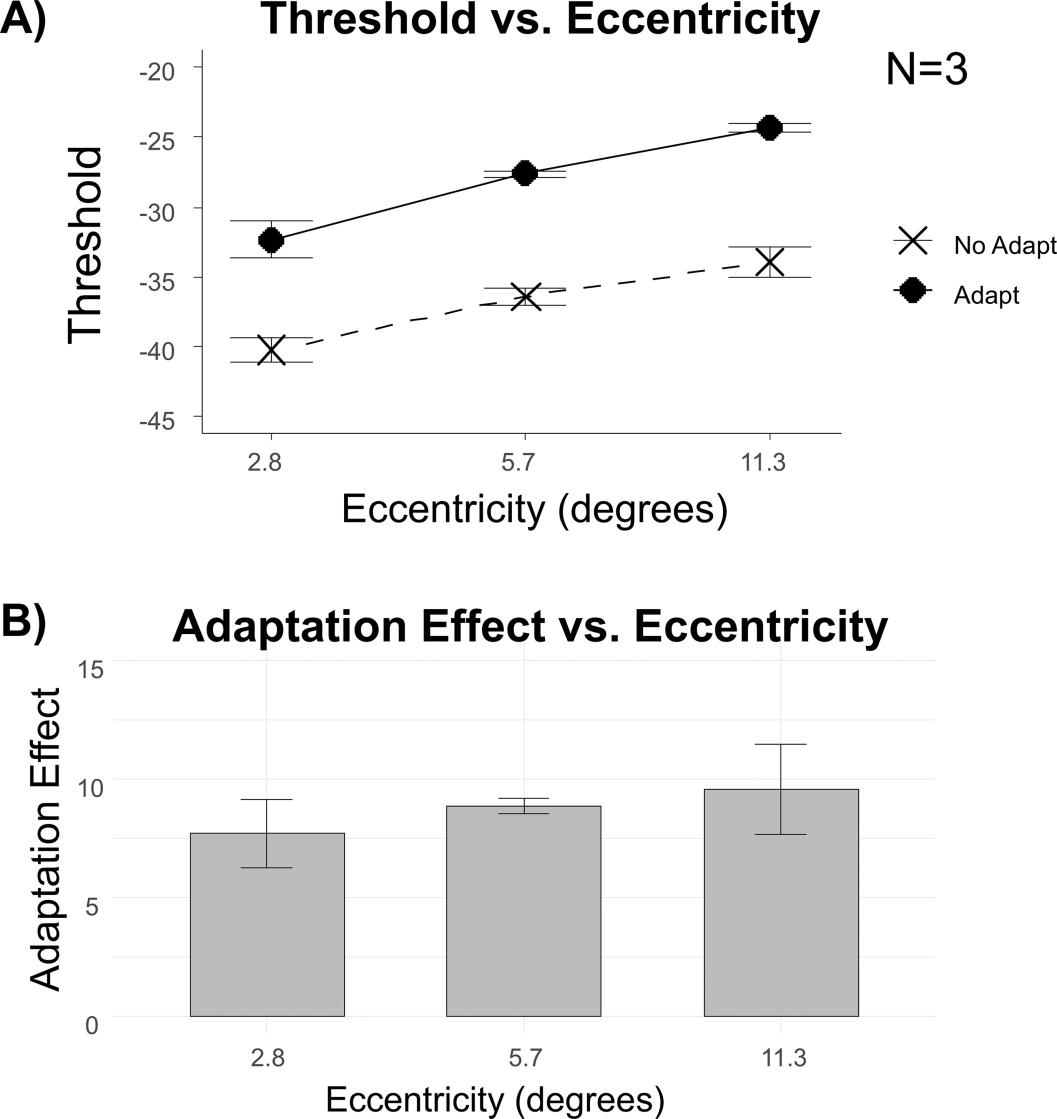
The effects of eccentricity and contour erasure on target detection threshold. In panel (**A**), the dashed line and crosses represent the data without contour erasure, and the continuous line and the diamonds represent the data with contour erasure. Panel (**B**) shows the adaptation effect (difference between the Adapt and No Adapt condition) across three eccentricity levels (log spacing). The error bars depict ±1 standard error of measurement.

Figure 2 shows the averaged data of three participants (P0, P1, and P2). The stimuli were presented at three eccentricities: 2.8 degrees, 5.7 degrees, and 11.3 degrees. The line plot in the upper panel shows the averaged threshold with adaptation (solid curve) and without adaptation (dashed curve) plotted against eccentricity. The lower panel represents the adaptation effect—that is, the elevation detection threshold (in decibels), across the three eccentric locations. The individual data are presented in the Supplementary Figure S1.

Target threshold increased after the contour was adapted and thresholds increased with increasing eccentricity. In general, detection thresholds following contour erasure were 5.69 to 12.7 dB (equivalent to a 1.92–4.33-fold increase) compared to the unadapted condition.

We performed a two-way repeated measurement ANOVA and calculated the difference of Bayesian Information Criterion (ΔBIC) for each term by comparing models with and without each main effect and the interaction term separately. Using the method described in Wagenmakers (2007; Equations 12 and 14), we estimated the posterior probability that the model including the effect of interest is more plausible.

There was strong evidence for a main effect of adaptation (*F*(1, 2) = 426.4, *p* = 0.002, η^2^= 0.795, ΔBIC = −2.636), and a significant main effect of eccentricity (*F*(2, 4 ) =7.95, *p* = 0.04, η^2^ = 0.637, and ΔBIC = −4.329). However, the interaction between eccentricity and adaptation was not significant (*F*(2, 4) = 0.29, *p* = 0.764, and ΔBIC = −0.003).

Experiment 1 examined whether the strength of the contour erasure varied with eccentricity. Although detection thresholds increased with eccentricity overall, the size of the adaptation effect remained constant across locations. This suggests that, unlike other forms of perceptual filling-in, the contour erasure effect does not depend on visual field eccentricity at least for the range of eccentricities tested, highlighting its distinct spatial characteristics.

### Experiment 2: Effect of adapter size (spatial factor 2)

**Figure 3.**
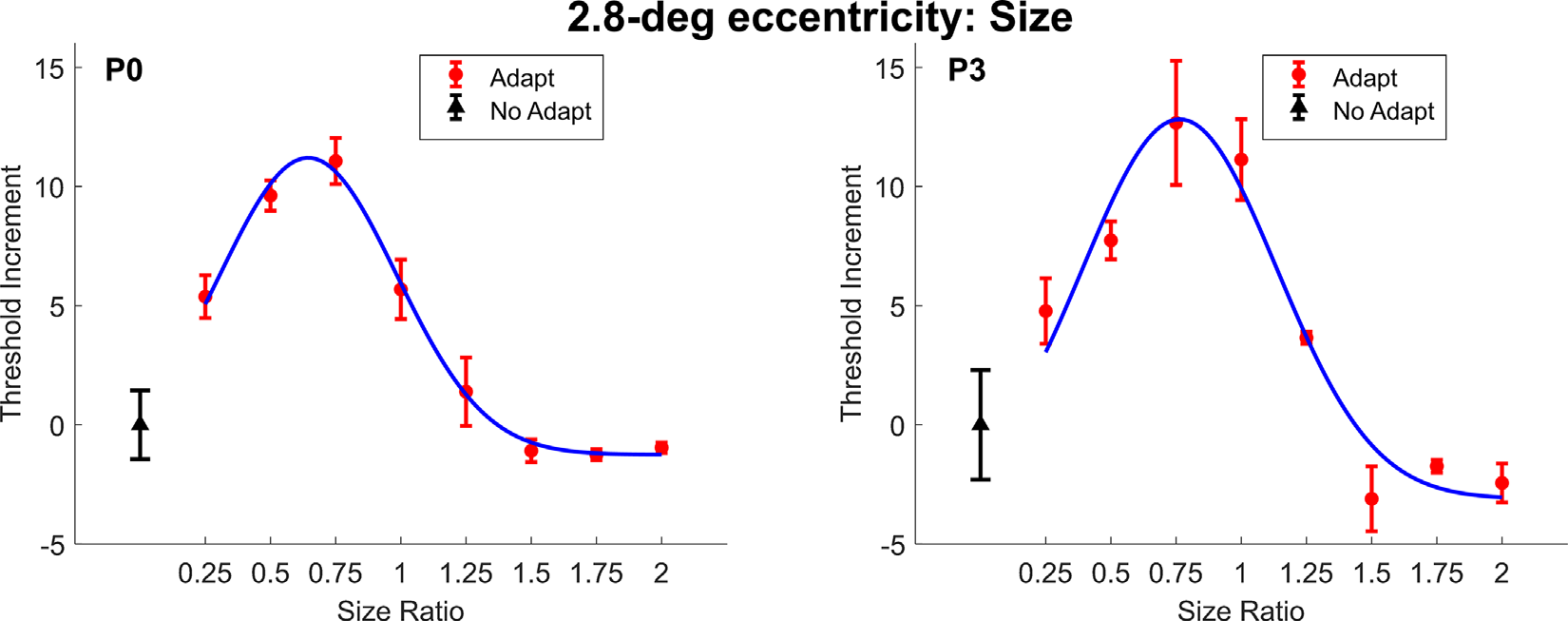
The threshold elevation is plotted as a function of adapter-to-target size ratio at 2.8-degrees eccentricity. The black triangle represents the data point without contour erasure. The red circles represent data points with contour erasure at different adapter sizes. Size ratio of one represents the condition when the adapter and target was of the same diameter. Size ratios larger than one represents the conditions when the adapter diameter was larger than that of the target, whereas size ratios smaller than one the conditions when the adapter diameter was smaller than that of the target. The blue smooth curve is the fitting result of a truncated Gaussian function. All error bars represent ±1 standard error of the measurements.

**Figure 4.**
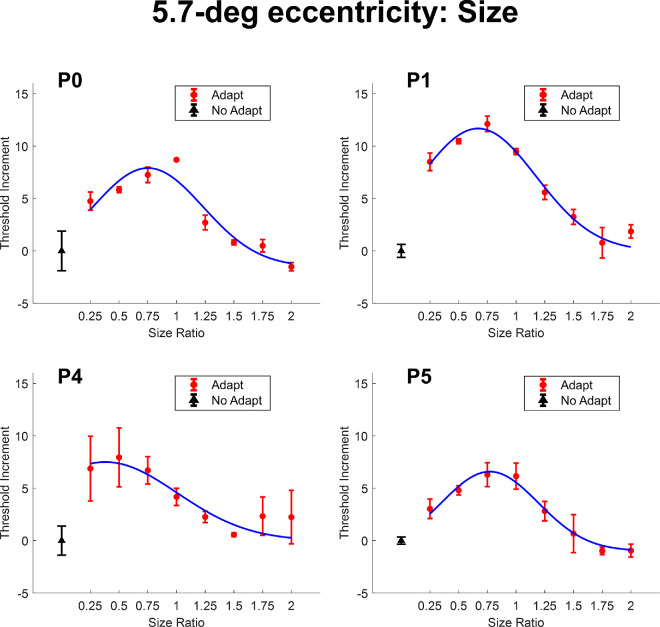
The threshold increment as a function of adapter-to-target size ratio at 5.7-degrees eccentricity. The black triangle represents the data point without contour erasure. The red disks represent data points with contour erasure at different adapter sizes. The blue smooth curve is the fitting result of a truncated Gaussian function. All error bars represent ±1 standard error of the measurements. Otherwise, as in Figure 3.

We defined adapter size as the ratio between the adapter diameter and that of the target. In this experiment, the size of the target remained constant, whereas the size of the adapter varied. A ratio of one represents the condition when the adapter and the target were of the same size. A ratio larger than one indicates that the adapter was larger than the target. A ratio smaller than one indicates that the adapter was smaller than the target. Adapter sizes ranged from 0.25 to 2 times the target diameter, corresponding to 0.5 degrees to 4 degrees of visual angle. Figure 3 presents the effect of changing adapter size on the target threshold at 2.8 degrees eccentricity (P0 and P3), whereas Figure 4 shows the results for 5.7 degrees eccentricity (P0, P1, P4. and P5).

To better visualize the adaptation effect, we subtracted the mean threshold values from the control condition (i.e. without adaptation) from those obtained under adaptation for each participant. Therefore, the value of the unadapted condition is always 0 in Figure 3 and all the following figures. A truncated Gaussian function was fitted to the data, defined as follows:

(1) $$\begin{array}{c} f\left(x\right)=a\times e^{\left(-\frac{{\left(x-\mu \right)}^{2}}{2\sigma^{2}}\right)},\; \end{array}$$

Where *f*(*x*), the threshold increment, is a function of *x*, the size ratio. Parameter *a*, a height parameter, μ, the center parameter, and σ, the width parameter were set as free parameters.

The results indicate that as the adapter size ratio deviated away from the same size as the target (1.0), the threshold elevation decreased. Surprisingly, the peak of the size tuning centered around 0.75 or less (*µ* values at 2.8 degrees: P0: 0.645, P3: 0.764; *µ* values at 5.7 degrees: P0: 0.752, P1: 0.669, P4: 0.380, P5: 0.771), suggesting that slightly smaller adapters increased the target threshold even more. Such an effect could be related to a combination of contour erasure effect and other masking or adaptation processes. The size tuning is wider at 5.7 degrees eccentricity (σ values: P0: 0.480, P1: 0.507, P4: 0.625, P5: 0.423) compared to 2.8 degrees eccentricity (σ values: P0: 0.339, P3: 0.372). To assess the differences between the threshold elevation distributions at the two eccentricity conditions (2.8 degrees and 5.7 degrees), we used a bootstrapping approach. For each eccentricity, we randomly sampled the participants’ data with replacement and fitted a truncated Gaussian model to estimate the sigma parameter for each bootstrap iteration. This process was repeated for 5000 iterations, producing two distributions of sigma estimates for the 2.8 degrees and 5.7 degrees eccentricity conditions. A Mann-Whitney *U* test showed a significant difference between the distributions for the two eccentricity conditions (U = 0, *p* < 0.001). We note that in the Mann–Whitney *U* test, a lower *U* value corresponds to a more pronounced difference between the groups and is associated with greater statistical significance. Such a result suggests that the sigma estimates for the 5.7 degrees condition were significantly larger than those for the 2.8 degrees condition.

These results demonstrate that the contour erasure effect is modulated by the spatial relationship between adapter and target size. Notably, the threshold elevation peaked when the adapter was slightly smaller than the target, suggesting that mechanisms beyond simple contour-specific adaptation may be involved. Furthermore, the broader tuning observed at greater eccentricities suggests that the spatial specificity of the effect decreases in peripheral vision.

### Experiment 3: Effect of adapter flicker rate (temporal factor 1)

**Figure 5.**
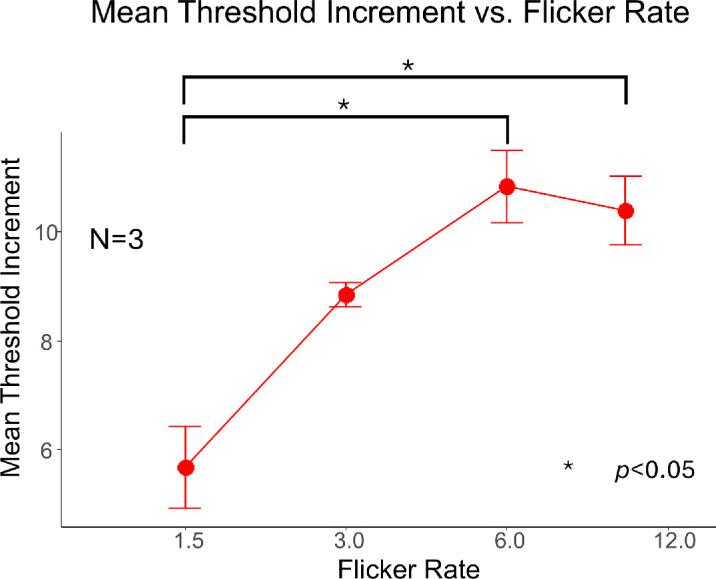
The effect of adapter flickering rate (in Hz) on target threshold at 5.7-degrees eccentricity. The error bars depict ±1 standard error of measurement. The mean results of three participants (P0, P1, and P2) are shown.

To test how the adapter flicker rate affects the adaptation magnitude, we manipulated the flicker rate of the adapter from 1.5 Hz to 10 Hz. The eccentricity was fixed at 5.7 degrees, and the adaptation duration at 3 seconds. Figure 5 shows the averaged threshold increments of the three participants (P0, P1, and P2) across different flickering rates. The individual data are presented in the Supplementary Figure S2. The threshold increment increased as the flickering rate increased from 1.5 Hz to 6 Hz, and then reached a plateau.

To examine the effect of the flickering rate on the contour erasure effect, we conducted a one-way repeated measurement ANOVA for the data from three participants. The result showed a significant flickering rate effect (*F*(3, 6) = 10.72, *p* = 0.008, η^2^ = 0.613, ΔBIC = −4.45). A post hoc analysis with Tukey's HSD method revealed that the threshold increment at 1.5 Hz was lower than the threshold increment at 6 Hz (*p_adjust_* = 0.009), and that 1.5 Hz increment was lower than the 10 Hz (*p_adjust_* = 0.013). No significant difference was found in the remaining pairwise comparisons. All the *p* values were controlled for the Family-Wise Error Rate (FWER).

These findings suggest that increasing the flicker rate of the adapter enhances the magnitude of contour erasure, particularly when comparing lower (1.5 Hz) to higher rates (≥6 Hz). However, the adaptation effect appears to saturate at approximately 6 Hz, indicating a potential limit to the rate-dependent sensitivity of the underlying mechanism. This pattern is consistent with the temporal characteristics of the transient visual channel, often associated with the magnocellular pathway, which is sensitive to low spatial frequency and high temporal frequency stimuli. The early increase and subsequent plateau in adaptation strength may reflect the frequency tuning properties of this pathway, suggesting that contour erasure could, at least in part, involve adaptation processes within transient, magnocellular-dominated circuits (Edwards, Goodhew, & Babcock, 2021; Kulikowski & Tolhurst, 1973; Masri, Grünert, & Martin, 2020).

### Experiment 4: Effect of adaptation duration (temporal factor 2)

**Figure 6.**
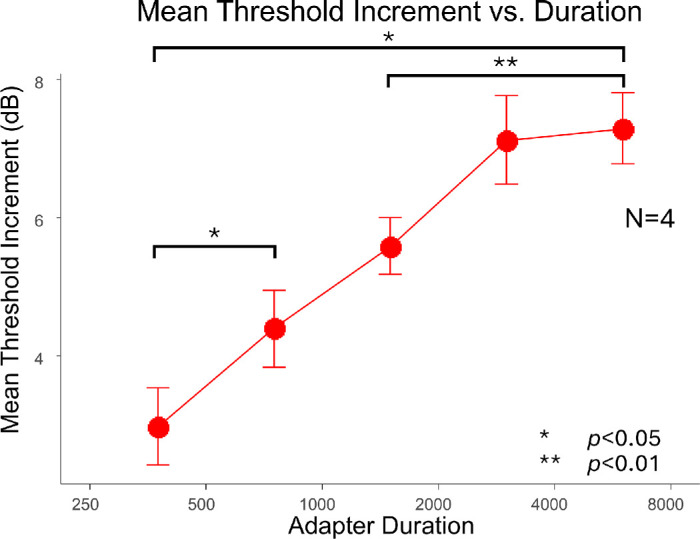
The effect of adaptation duration (in ms) on threshold elevation at 5.7-degrees eccentricity. The error bars depict ±1 standard error of measurement.

Figure 6 shows the effect of adaptation duration, ranging from 375 ms to 6000 ms, on the averaged threshold elevation across four participants (P0, P1, P4, and P5). The individual data are presented in the Supplementary Figure S3. In general, the threshold elevation increased with longer adaptation durations, consistent with previous findings (Greenlee et al., 1991). To statistically assess this effect, we conducted a one-way repeated measures ANOVA, which revealed a significant main effect of adaptation duration (*F*(4, 12) = 11.156, *p* = 0.040, η² = 0.788, ΔBIC = –4.820). The post hoc pairwise analysis with Tukey's HSD method showed three significant differences: threshold elevation at 375 ms was significantly lower than at 750 ms (*p_adjust_* = 0.027) and at 6000 ms (*p_adjust_* = 0.025), and threshold elevation at 1500 ms was significantly lower than at 6000 ms (*p_adjust_* = 0.002).

These results suggest that longer adaptation durations generally lead to stronger contour erasure effect.

### Experiment 5: Effect of interstimulus-interval (temporal factor 3)

**Figure 7.**
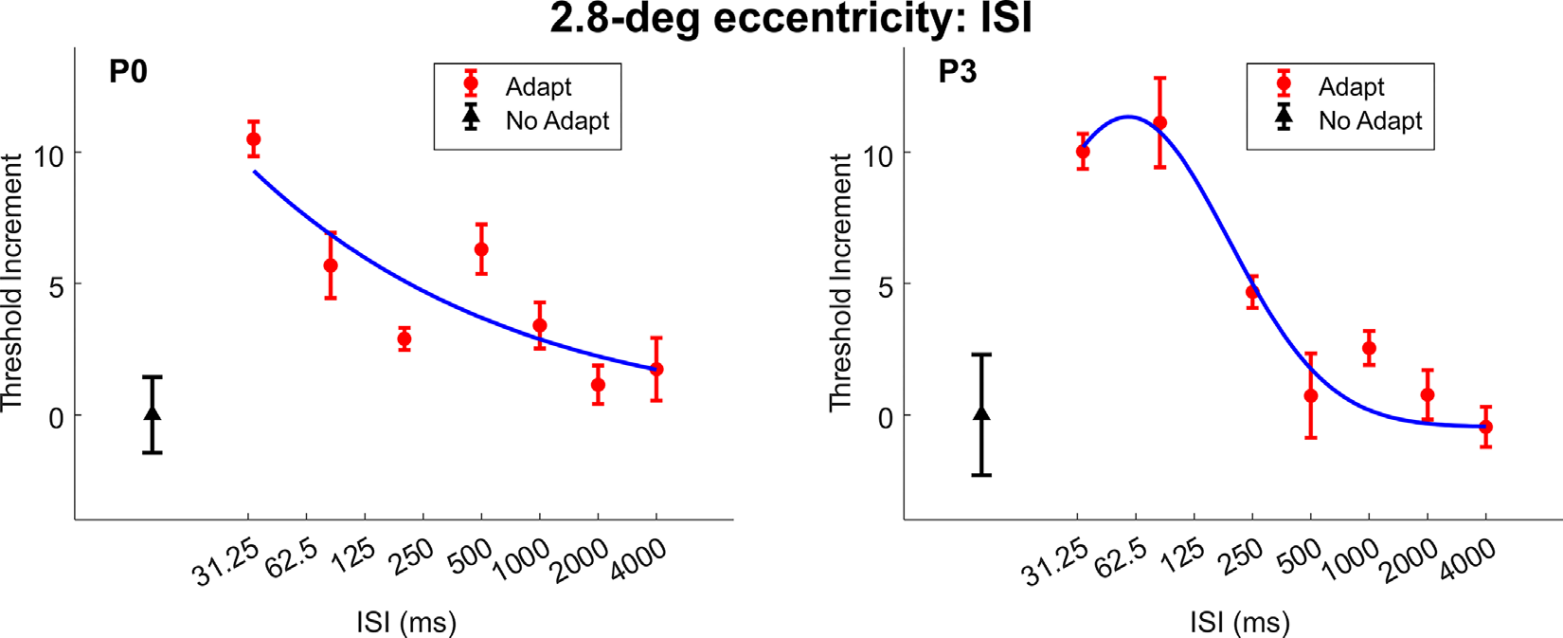
The target threshold increment data across different ISI values at 2.8-degrees. The black triangle represents the control condition without adaptation for each participant. The red disk markers represent the threshold increment data for different ISI values. The blue solid curve represents the truncated Gaussian function fitted to the threshold increment data. All error bars depict the standard error of the measurement. The data of P0 at 2.8-degrees is ill-constrained, therefore we use the model estimate at 33 ms as peak and then find the half value as the half recovery time.

**Figure 8.**
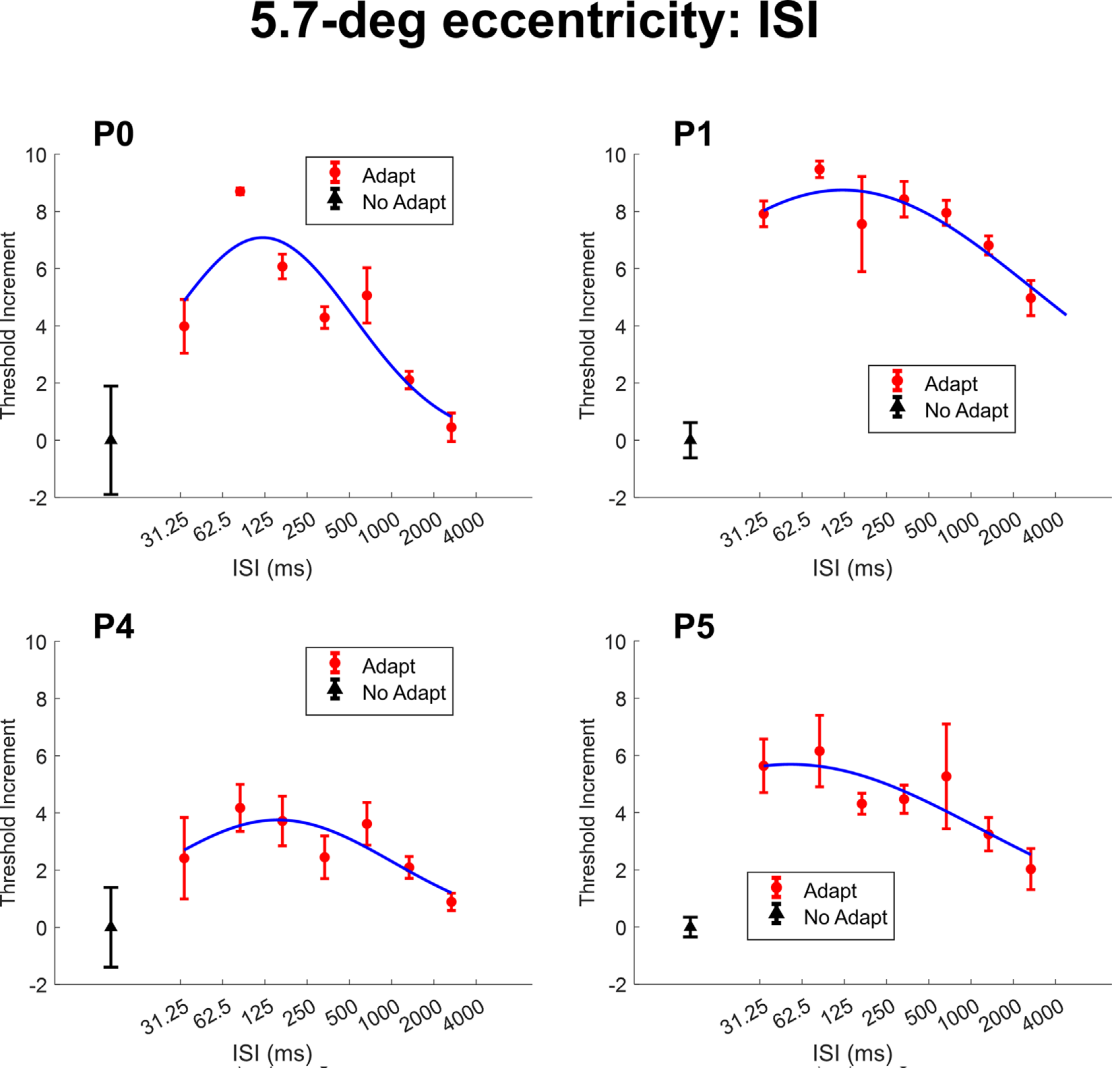
The target threshold increment data across different ISI values at an eccentricity of 5.7 degrees. The black triangle represents the no adaptation condition for each participant. The red disk markers represent the threshold elevation data as a function of recovery time (i.e. different ISI values). The blue solid curve represents the truncated Gaussian function fitted to the threshold increment data. All error bars depict the standard error of the measurement.

We next investigated the recovery dynamics of contour erasure by varying the ISI between the adapter and the target. The results are shown in Figure 7 for para-central locations (2.8 degrees eccentricity; P0 and P3) and in Figure 8 for more peripheral locations (5.7 degrees eccentricity; P0, P1, P4, and P5), where threshold increments are plotted against different ISIs (logarithmically spaced).

At both eccentricities, the threshold increment initially increased with ISI, reaching a peak, and then gradually decreased. To quantify this pattern, we fitted a truncated Gaussian model (see Equation 1) to each participant's data set. We estimated the 50% recovery time as the ISI corresponding to half of the maximum threshold increment predicted by the fitted function.

At all eccentricities, the contour erasure effect first increased and then diminished with ISI, forming an inverted-U shaped pattern across time, with a peak between 80 and 100 ms ISI.

For the 2.8 degrees eccentricity condition, the half-recovery times ranged from 231 ms to 258 ms, suggesting relatively fast recovery. In contrast, for the 5.7 degrees eccentricity condition, recovery was slower, with half-recovery times ranging from 688 ms to 4780 ms across participants. Such a difference caused by eccentricity suggests that the recovery rate is slower with higher eccentricity.

## Discussion

The current study aimed to characterize the spatial and temporal properties of the contour erasure effect. Such information allows us to compare the contour erasure quantitatively and with other visual phenomena, such as contrast adaptation, artificial scotoma filling-in, and metacontrast, and leads to a better understanding of the underlying mechanisms. We conducted five experiments that systematically manipulated two spatial factors—eccentricity and adapter size—and three temporal factors—adapter flicker rate, adaptation duration, and the ISI between adapter and target.

In experiment 1, we found that whereas target detection thresholds increased with eccentricity, the magnitude of the contour erasure effect—indexed by threshold elevation relative to baseline—remained constant across different eccentricities. This finding is particularly noteworthy when contrasted with artificial scotoma filling-in, which is known to be stronger in the visual periphery (De Weerd et al., 1998). The result suggests that contour erasure does not simply reflect a passive loss of spatial sensitivity in the periphery, but rather arises from a different, potentially more uniform mechanism across the visual field.

In experiment 2, we showed that the contour erasure effect was strongest when the adapter was slightly smaller than the target, rather than matching the target contour exactly. This spatial offset might suggest that contour erasure is not mediated by simple local mechanisms such as edge or border detectors, but rather involves spatially extended processes, possibly through horizontal connections in early visual cortex (Chen, Kasamatsu, Polat, & Norcia, 2001; Stettler, Das, Bennett, & Gilbert., 2002) or feedback from higher-order visual areas (Angelucci, Levitt, & Lund, 2002; Angelucci et al., 2002). These two neural signals could modulate activity beyond the directly stimulated regions. Another possibility is that neurons with larger receptive fields encompass both the adapter and target edge regions, thereby enhancing the adaptation effect. However, a detailed analysis of the underlying mechanisms of such additional effects are beyond the scope of our current study. Future research could focus on separating these effects to further distinguish the processes involved.

In experiment 3, we manipulated the flickering rate of the adapter and found that threshold elevation increased from 1.5 to 3 Hz and then plateaued at approximately 6 Hz. This response profile aligns with the known temporal tuning of the magnocellular (M) pathway, which is sensitive to low spatial and high temporal frequencies (Lennie, 1980; Merigan & Maunsell, 1993). The involvement of the transient visual pathway is further supported by previous studies showing that flicker adaptation can bias the apparent spatial frequency (Kaneko et al., 2015) or improve visual acuity by reducing crowding (Arnold, Williams, Phipps, & Goodale, 2016; Tagoh, Hamm, Schwarzkopf, & Dakin, 2024). Together, our findings suggest that contour erasure is strongly modulated by transient input and is likely mediated, at least in part, by the M-pathway.

In experiment 4, we examined the effect of adaptation duration and found that threshold elevation increased with longer durations, consistent with other adaptation phenomena such as contrast adaptation (Greenlee et al., 1991). Importantly, however, the contour erasure effect was clearly measurable even at durations as short as 375 ms, which contrasts sharply with artificial scotoma filling-in that typically requires sustained adaptation of 4 to 6 seconds or more (De Weerd, Gattass, Desimone, & Ungerleider, 1995; De Weerd et al., 1998). This again points to a distinct underlying mechanism for contour erasure—one that is more rapidly engaged.

In dxperiment 5, we varied the interstimulus interval between the adapter and the target and observed an inverted-U-shaped relationship, where threshold elevation peaked at ISIs around 80–100 ms and then declined. This temporal pattern resembles those observed in backward masking (Weisstein & Haber, 1965) and metacontrast (Breitmeyer, 1978; Breitmeyer & Ganz, 1976), suggesting that contour erasure follows a fast, transient time course. However, the temporal resolution of the current study was limited by the monitor 60 Hz refresh rate and the relatively sparse sampling of short ISIs. Therefore, to make stronger conclusions of the shared underlying mechanisms with other masking effects, further research with a better temporal resolution apparatus would be needed. The estimated half-recovery times (231–258 ms for parafoveal stimuli and up to 4.8 seconds for peripheral stimuli) further support the idea that this effect is driven by a short-lived adaptation mechanism.

Together, these results suggest that contour erasure is driven by a fast-acting, spatially extended adaptation mechanism in early visual areas. Its temporal dynamics and sensitivity to flickering stimulation point to the transient M-pathway as a likely contributor (Lennie, 1980; Merigan & Maunsell, 1993). The spatial offset and extended influence zone of the adapter suggest non-local interactions, possibly mediated by horizontal connections in V1 (Angelucci & Bressloff, 2006; Angelucci et al., 2002; Stettler et al., 2002) or feedback from higher extrastriate areas (Gilbert & Li, 2013; Nassi & Callaway, 2009).

Our results also provide a more objective, threshold-based measure of contour erasure, as opposed to subjective visibility ratings used in previous studies (Cox, Lowe, Blake, & Maier, 2014). This helps reduce potential bias and allows finer comparison with other visual phenomena. The rapid onset and short-lived nature of the contour erasure effect distinguish it from other adaptation effects such as artificial scotoma filling-in and make it a potentially valuable tool for probing fast dynamics in early visual processing.

Future directions may include neuroimaging or electrophysiological studies to localize the cortical areas involved in contour erasure and to test the involvement of the M-pathway more directly. Another promising line of research would be to explore how contour erasure interacts with perceptual grouping or figure-ground segregation, and whether it facilitates perceptual filling-in from the surround as proposed by De Weerd et al. (1998). As the shared mechanisms underlying contour erasure, contrast adaptation and perceptual filling-in remain unclear, yet another future direction could focus on investigating the common processes involved in the three closely related phenomena. Finally, understanding how contour erasure affects real-world object perception, particularly under dynamic viewing conditions, could extend its relevance beyond basic vision science.

## Conclusions

The current study examined the spatiotemporal characteristics of the contour erasure effect through five experiments, aiming to understand its underlying mechanisms and how it differs from related phenomena like contrast adaptation, artificial scotoma filling-in, and meta-contrast masking. Across two spatial manipulations—eccentricity and adapter size—and three temporal parameters—adapter flickering rate, adapter duration, and inter-stimulus interval—we found that contour erasure is a rapid and spatially extended adaptation effect likely originating in early visual areas.

Unlike artificial scotoma filling-in, which is stronger in peripheral vision, contour erasure remained constant across eccentricities, suggesting a distinct mechanism. The effect was evident even after brief adaptation (375 ms), and it decayed rapidly with a half-life of approximately 250 ms, consistent with cortical pattern adaptation. Contour erasure was enhanced by higher adapter flickering rates, saturating around 6 Hz, pointing toward an involvement of the magnocellular (transient) visual pathway. Moreover, the maximal effect occurred not when the adapter and target contours were spatially superimposed, but when they were offset by a small spatial distance, highlighting the role of non-local interactions—potentially through horizontal connections in V1 or feedback from higher-order visual areas.

Together, these findings indicate that contour erasure arises from a short-lived, spatially extended adaptation mechanism, one that is temporally tuned and sensitive to transient stimulation. Our results contribute to a more precise understanding of contour erasure in visual processing and offer a quantitative, objective approach to studying this phenomenon—complementing previous studies that relied on subjective measures. This work also paves the way for future investigations into the neural basis of dynamic visual adaptation and boundary perception.

## Supplementary Material

Supplement 1

Supplement 2

Supplement 3

Supplement 4
