# Supplementary figures and images for: The spatial and temporal properties of the contour erasure effect and perceptual filling-in
Source: J Vis. 2025 Dec 11;25(14):4. doi: 10.1167/jov.25.14.4 (PMC12704214; doi:10.1167/jov.25.14.4)

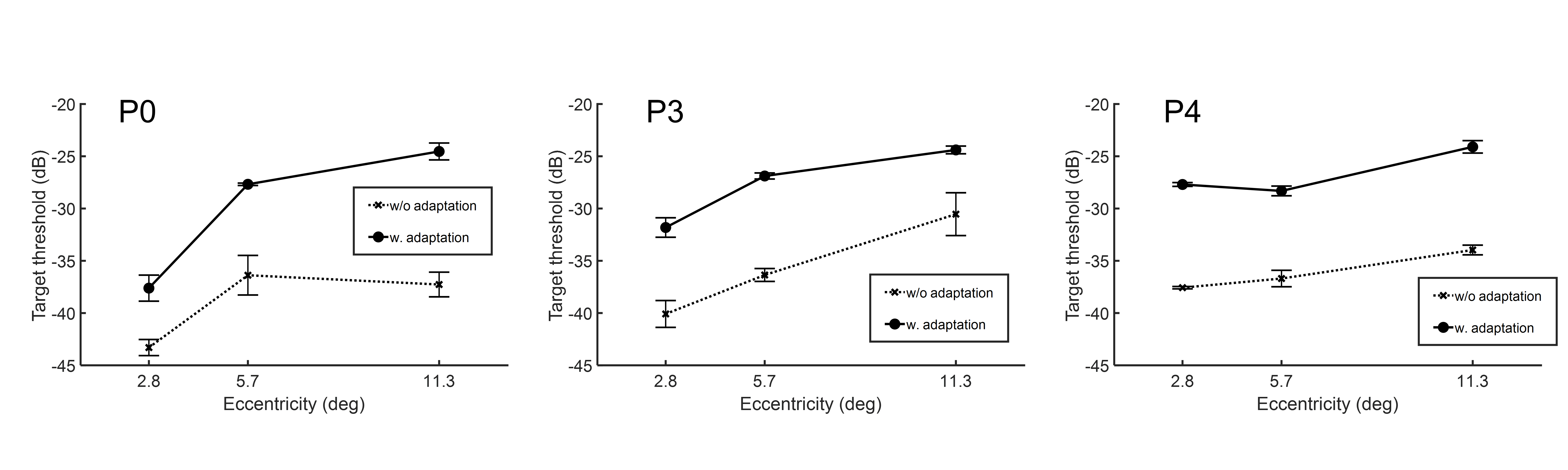

Supplement: Supplement 1 [file jovi-25-14-4_s001.tif]

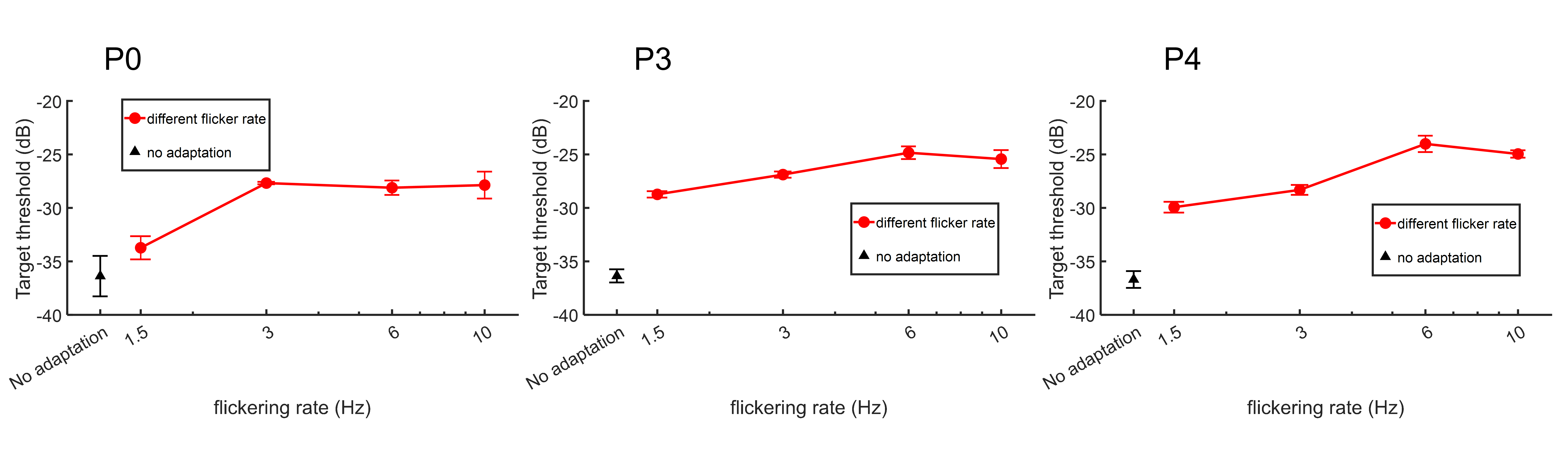

Supplement: Supplement 2 [file jovi-25-14-4_s002.tif]

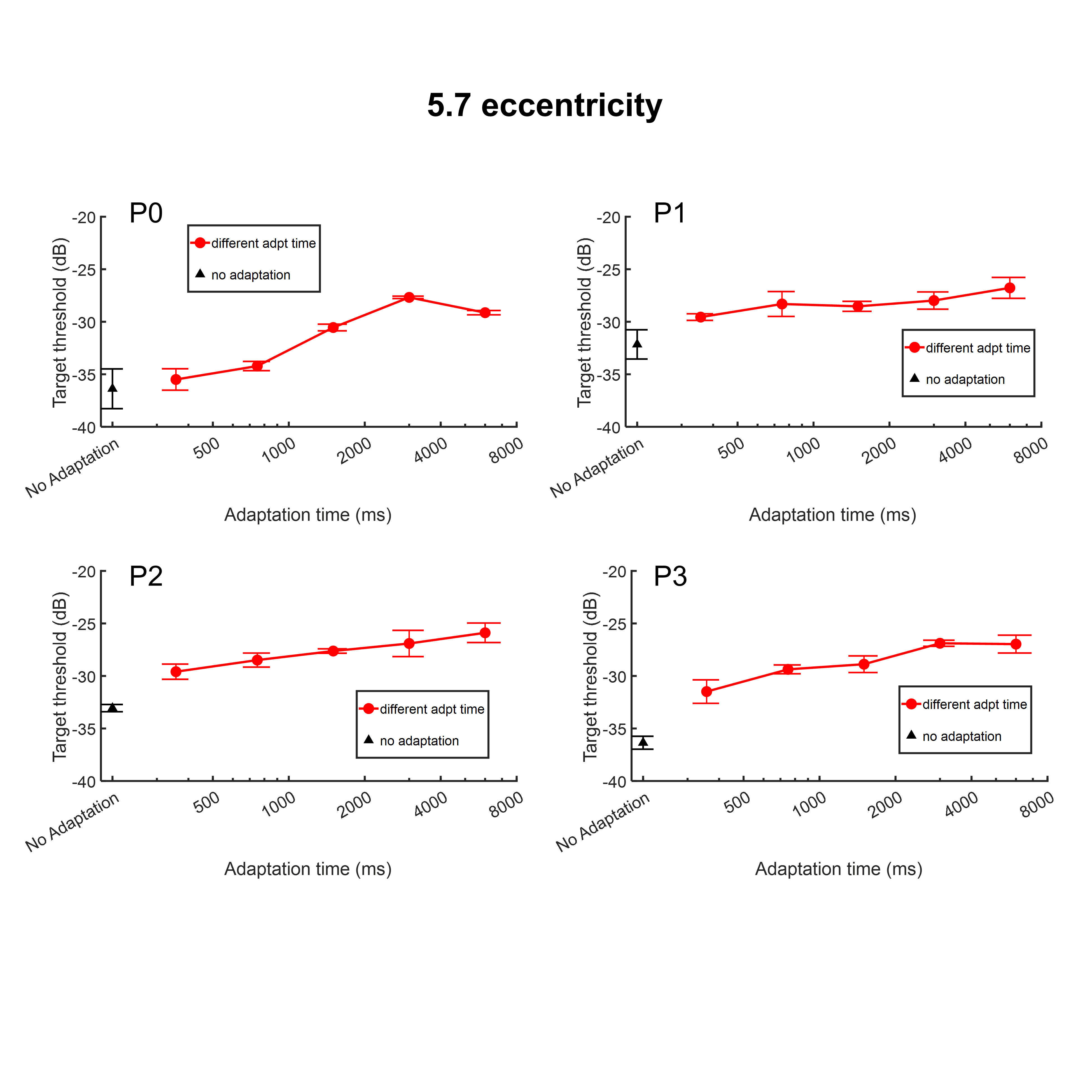

Supplement: Supplement 3 [file jovi-25-14-4_s003.tif]
